# Non-canonical Keap1-independent activation of Nrf2 in astrocytes by mild oxidative stress

**DOI:** 10.1016/j.redox.2021.102158

**Published:** 2021-10-02

**Authors:** Bashayer R. Al-Mubarak, Karen F.S. Bell, Sudhir Chowdhry, Paul J. Meakin, Paul S. Baxter, Sean McKay, Owen Dando, Michael L.J. Ashford, Irina Gazaryan, John D. Hayes, Giles E. Hardingham

**Affiliations:** a Centre for Discovery Brain Sciences, University of Edinburgh, Hugh Robson Building, George Square, Edinburgh, EH8 9XD, UK; b Biomedical Research Institute, University of Dundee, Ninewells Hospital and Medical School, Dundee, DD1 9SY, UK; c UK Dementia Research Institute at the University of Edinburgh, Chancellor's Building, Edinburgh Medical School, EH16 4SB, UK; d Department of Chemistry and Physical Sciences, Dyson College of Arts and Sciences, Pace University, Pleasantville, NY, 10570, USA; e Discovery & Translational Science Department, Leeds Institute of Cardiovascular and Metabolic Medicine, University of Leeds, LS2 9JT, UK; f Division of Systems Medicine, School of Medicine, University of Dundee, Ninewells Hospital & Medical School, Dundee, UK; g Behavioral Genetics Unit, Department of Genetics, King Faisal Specialist Hospital and Research Center, P.O Box 3354, Riyadh, 11211, Saudi Arabia

**Keywords:** Astrocytes, Gene transcription, NRF2, Keap1, Oxidative stress, Neurodegeneration

## Abstract

The transcription factor Nrf2 is a stress-responsive master regulator of antioxidant, detoxification and proteostasis genes. In astrocytes, Nrf2-dependent gene expression drives cell-autonomous cytoprotection and also non-cell-autonomous protection of nearby neurons, and can ameliorate pathology in several acute and chronic neurological disorders associated with oxidative stress. However, the value of astrocytic Nrf2 as a therapeutic target depends in part on whether Nrf2 activation by disease-associated oxidative stress occludes the effect of any Nrf2-activating drug. Nrf2 activation classically involves the inhibition of interactions between Nrf2's Neh2 domain and Keap1, which directs Nrf2 degradation. Keap1 inhibition is mediated by the modification of cysteine residues on Keap1, and can be triggered by electrophilic small molecules such as tBHQ. Here we show that astrocytic Nrf2 activation by oxidative stress involves Keap1-independent non-canonical signaling. Keap1 deficiency elevates basal Nrf2 target gene expression in astrocytes and occludes the effects of tBHQ, oxidative stress still induced strong Nrf2-dependent gene expression in Keap1-deficient astrocytes. Moreover, while tBHQ prevented protein degradation mediated via Nrf2's Neh2 domain, oxidative stress did not, consistent with a Keap1-independent mechanism. Moreover the effects of oxidative stress and tBHQ on Nrf2 target gene expression are additive, not occlusive. Mechanistically, oxidative stress enhances the transactivation potential of Nrf2's Neh5 domain in a manner dependent on its Cys-191 residue. Thus, astrocytic Nrf2 activation by oxidative stress involves Keap1-independent non-canonical signaling, meaning that further Nrf2 activation by Keap1-inhibiting drugs may be a viable therapeutic strategy.

## Graphical abstract

**Figure:**
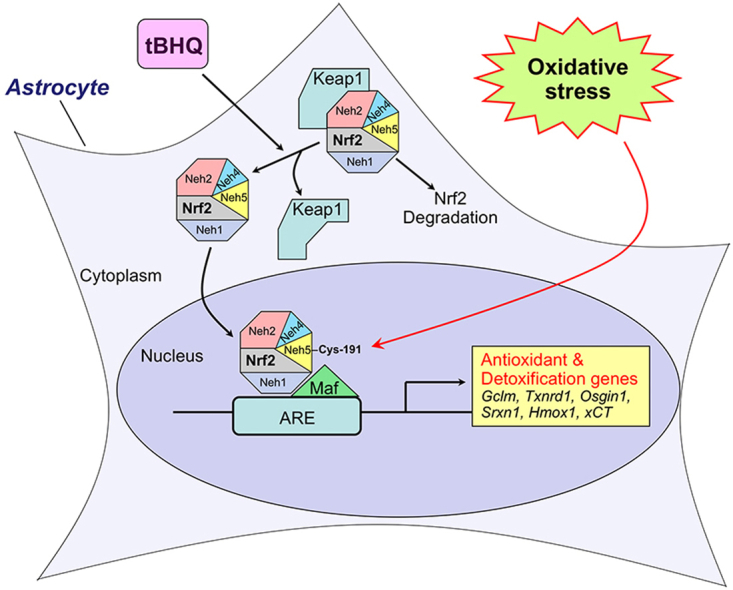

## 1 Introduction

Animals have developed adaptive, protective defence programs mediated by de novo gene expression to protect against oxidative stress. Key among them are those genes operating under the control of antioxidant response elements (AREs) within their *cis*-acting promoter regions [1,2]. ARE's are bound by the transcription factor Nrf2 (encoded by *Nfe2l2*) in a complex with small Maf proteins. The key point of regulatory control is the presence of Nrf2 in the nucleus, under regulation by Nrf2's inhibitor Kelch-like ECH-associated protein 1 (Keap1) [3,4]. Under un-stressed conditions, steady state Nrf2 levels are very low, as Keap1 interacts with Nrf2 primarily via its Neh2 domain and targets it for ubiquitin mediated degradation [5]. However, a variety of stressors acting on Keap1's redox-sensitive cysteine residues interfere with its inhibition of Nrf2, allowing it to accumulate, enter the nucleus, and drive ARE-mediated gene expression, which include antioxidant, detoxification and proteostasis genes.

Nrf2 plays an important role in influencing the trajectory of neurodegenerative and neurological disease. Nrf2 deficient mice exacerbate pathology in models of AD, PD, Huntington's disease, vascular impairment/stroke and multiple sclerosis, while genetic or pharmacological activation of Nrf2 has shown beneficial effects in these models through its capacity to attenuate various pathological processes such as neuroinflammation, mitochondrial dysfunction and oxidative stress [[6], [7], [8]]. In the CNS, astrocytes are a key site for Nrf2 activation, evidenced by early reporter gene studies [[9], [10], [11]]. In contrast, the Nrf2 pathway has negligible activity in forebrain neurons due to epigenetic repression of the Nrf2 gene promoter in early development [12] and has abnormally high instability of what little protein is made [13]. Nevertheless, Nrf2 activation in astrocytes, either via small molecular Keap1 inhibitors, or astrocyte-specific overexpression not only protects the astrocytes themselves, but also protects nearby neurons in vitro by a mechanism at least in part due to the production and release of the antioxidant glutathione [10,14,15]. This non-cell-autonomous neuroprotective effect is also observed in mice over-expressing Nrf2 subjected to models of ALS, PD, stroke and hypo-perfusion [6,8].

Although these experiments show the importance of astrocytic Nrf2 for brain health and as a therapeutic target, they do not address how Nrf2 is activated by endogenous stresses, particularly mild oxidative stress, which activates Nrf2 dependent gene expression in astrocytes and likely a contributor to Nrf2 activation of endogenous Nrf2 in response to stresses such as inflammation and ischemia/reperfusion (in which it has a neuroprotective effect [16,17]). A key issue for the therapeutic targeting of Nrf2 in astrocytes (e.g. via small molecules) in neurological/neurodegenerative diseases associated with oxidative stress is whether such stress has already activated Nrf2 via Keap1 inhibition, thus occluding the effect of any therapeutic intervention. Surprisingly, we found that activation of Nrf2-dependent gene expression in astrocytes by mild oxidative stress occurs via a non-canonical Keap1-independent pathway in contrast to pharmacological Keap1 inhibitors, rendering such compounds effective even under conditions of oxidative stress.

## 2 Results

In mixed cultures of cortical neurons and astrocytes, non-toxic (≤100 μM, Fig. S1a) concentrations of H_2_O_2_ induce classical Nrf2 target genes *Srxn1* and *Hmox1* and *Slc7a11* in WT but not Nrf2^−/−^ cultures ([16], confirmed in Fig. S1b), showing that no Nrf2-independent pathway is active in controlling these genes in the context of H_2_O_2_ treatment. Moreover, astrocytes are the only contributor to Nrf2 responses in mixed cultures, because Nrf2 expression is repressed in neurons to the extent that they are unable to mediate Nrf2-dependent gene expression [12,16]. As such, neither the electrophilic Keap1 inhibitor tBHQ nor H_2_O_2_, can trigger induction of these Nrf2 target genes, in contrast to the robust response of neuron-free astrocyte cultures [12,16].

**Fig. 1:**
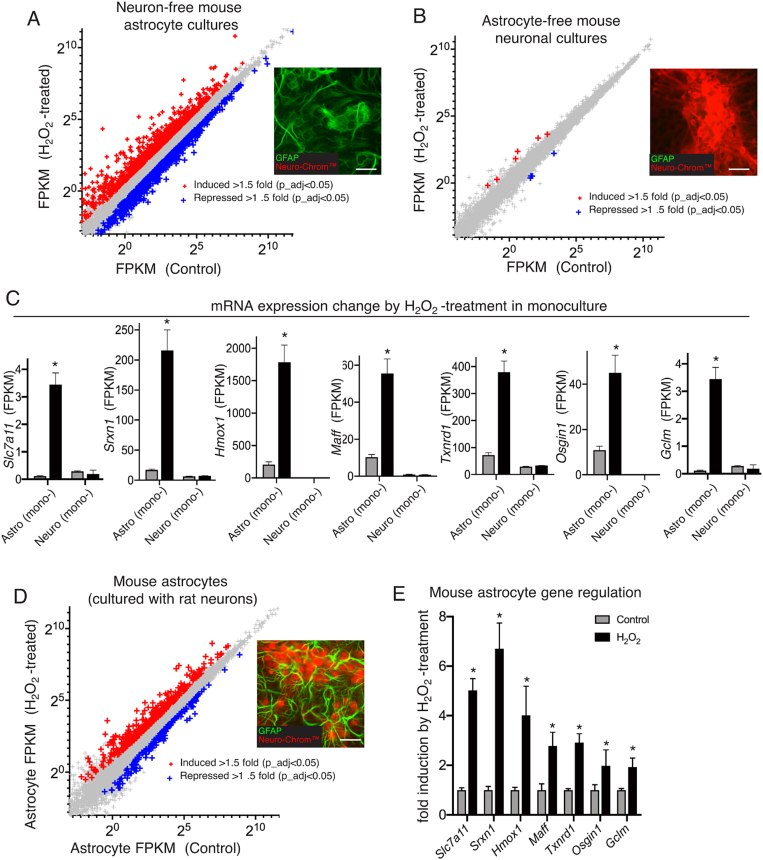
Oxidative stress induces Nrf2 target genes in astrocytes but not neurons. **A,B)** Neuron-free astrocyte cultures (A) and astrocyte-free neuronal cultures (B) were exposed to H_2_O_2_ for 4h after which RNA was harvested and RNA-seq performed. Genes significantly up- (red) and down- (blue) -regulated >1.5 fold (DESeq2 Benjamini Hochberg-corrected p-value <0.05) are shown (n = 3). Insets show pictures of immunofluorescent staining using an astrocyte-specific antibody (GFAP) and a neuron-specific antibody cocktail (Neurochrom). Both antibodies were used on both cultures to confirm their purity. **C)** A comparison of the expression (FPKM, Fragments Per Kilobase of transcript per Million mapped reads) of seven known Nrf2 target genes in neuron-free astrocyte cultures vs. astrocyte-free neuronal cultures ± H_2_O_2_. * Benjamini Hochberg-corrected p-value <0.05 (control vs. H_2_O_2_). **D)** Mouse astrocytes were co-cultured with rat neurons, and treated as in (A) and (B) ± H_2_O_2_ and subjected to RNA-seq. We sorted the mouse (astrocyte) reads using our Python tool *Sargasso* [20] and analyzed differentially expressed genes as in (A) and (B). Inset illustrates the mixed culture of astrocytes and neurons. **E)** For genes shown in (C), the fold change in co-cultured astrocytes is shown. * Benjamini Hochberg-corrected p-value <0.05 Con vs. H_2_O_2._ (For interpretation of the references to colour in this figure legend, the reader is referred to the Web version of this article.)

Genome-wide analysis of responses to non-toxic doses of H_2_O_2_ in mouse cortical astrocyte cultures (neuron-free) and astrocyte-free neuronal cultures revealed that H_2_O_2_ induced a number of known Nrf2 target genes >2-fold in astrocytes including *Srxn1* and *Hmox1* and *Slc7a11* but also *Txnrd1*, *Maff*, *Osgin1*, and *Gclm* (Fig. 1a,c). We also performed a systematic analysis of induction of Nrf2 target genes, defined in a comprehensive study [18] as those whose promoters contain a ChIP-seq peak and are either downregulated in Nrf2-deficient MEFs (Nrf2^+/+^ vs. Nrf2^−/−^) or up-regulated in Keap1-deficient MEFs (Keap1^+/+^ vs. Keap1^−/−^). 41 Nrf2 target genes as defined by this criteria were induced >2-fold in astrocytes (Table S1), but none of the 41 were induced in neurons (Fig. 1b and c, Table S1). Given that astrocytes are the only contributor to Nrf2 responses in neuron/astrocyte mixed cultures, we wanted to use this preparation for subsequent experiments, applying doses of H_2_O_2_ that were not toxic to either neurons or astrocytes in the culture. The rationale behind this is that astrocytes most resemble an *in vivo* morphology and transcriptome when co-cultured with neurons [19] and so are best studied in the presence of neurons, and also their expression levels are higher than in neurons both basally and even more so post- H_2_O_2_ treatment, so the astrocyte expression dominates. To further confirm that mouse astrocytes respond appropriately to H_2_O_2_ when in co-culture we cultured mouse neurons with rat astrocytes, and employed our Python tool *Sargasso* which enables species-specific sorting of bulk RNA-seq reads in silico [20], thus profiling the (mouse) astrocytic response in the co-culture (Fig. 1d). Indeed, all the H_2_O_2_-induced Nrf2 target genes in Fig. 1c are induced in astrocytes when in co-culture (Figs. 1e), and 34 out of the larger set of 41 Nrf2 target genes induced, and 1 other Nrf2 target gene (*Fgd*) was induced >2-fold in the co-cultured astrocytes but not induced in the astrocyte mono-culture (Table S1), though none were induced in astrocyte-free neuronal cultures (Table S1). We therefore studied astrocytic Nrf2-dependent gene expression in the context of mixed neuron/astrocyte cultures, focusing on *Srxn1*, *Hmox1* and *Slc7a11*, where the level of expression in astrocytes dominates a mixed culture of neurons and astrocytes.

**Fig. 2:**
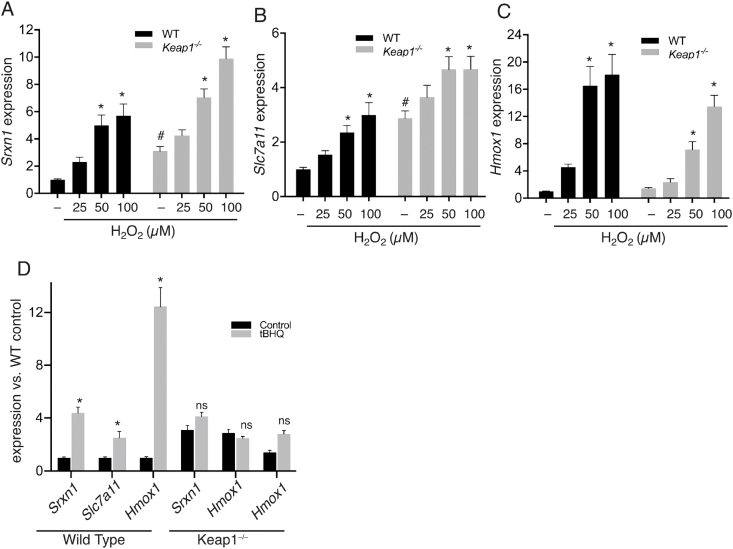
Mild oxidative stress induces Nrf2-regulated gene expression in Keap1^−/−^ cultures. **A-C)** Mixed astrocyte/neuronal cultures of the indicated genotypes were treated with the indicated doses of H_2_O_2_ for 6h after which expression of *Srxn1* (A), *Slc7a11* (B) and *Hmox1* (C) was assessed by qRT-PCR and normalized to *Gapdh*. Expression levels presented herein relative to WT-control. *P < 0.05 two-way ANOVA with Tukey's *post-hoc* test, ^#^ effect of genotype on basal levels (two-way ANOVA with Sidak's *post-hoc* test, ^#^*P* < 0.05 (n = 5–6). D) Mixed astrocyte/neuronal cultures of the indicated genotypes were treated with tBHQ for 6h after which gene expression was assessed as in A-C. *P < 0.05, (compared with WT-control), two-way ANOVA with Tukey's *post-hoc* test, (n = 4–6).

We hypothesized that H_2_O_2_ activates Nrf2-dependent gene expression in astrocytes via the canonical pathway of inhibition of Keap1-mediated Nrf2 degradation, and should therefore be occluded by Keap1 deficiency. Basal expression of *Srxn1*, and *Slc7a11* were increased in Keap1^−/−^ cultures (as expected), and *Hmox1* was much more modestly affected (likely to due to a negative feedback of constitutive Nrf2 activating signal on the *Hmox1* promoter). Strikingly, H_2_O_2_ further induced expression of *Srxn1*, and *Slc7a11* and *Hmox1* (Fig. 2a–c). In contrast, classical Nrf2 activator tBHQ induced *Srxn1*, *Hmox1* and *Slc7a11* in WT but not in Keap1^−/−^ cultures (Fig. 2d), consistent with tBHQ's mechanism of action as a Keap1 inhibitor [[21], [22], [23]]. Thus, in contrast to tBHQ, mild oxidative stress induces Nrf2-dependent gene expression in astrocytes via a Keap1-independent mechanism. We also confirmed that neuron-free astrocyte mono-cultures respond similarly to H_2_O_2_: *Srxn1*, and *Slc7a11* and *Hmox1* were all induced by H_2_O_2_ (Fig. S2a), their basal expression was elevated in Keap1^−/−^ cultures vs. WT (Fig. S2b), and this expression was further induced by H_2_O_2_ despite the absence of Keap1 (Fig. S2c).

**Fig. 3:**
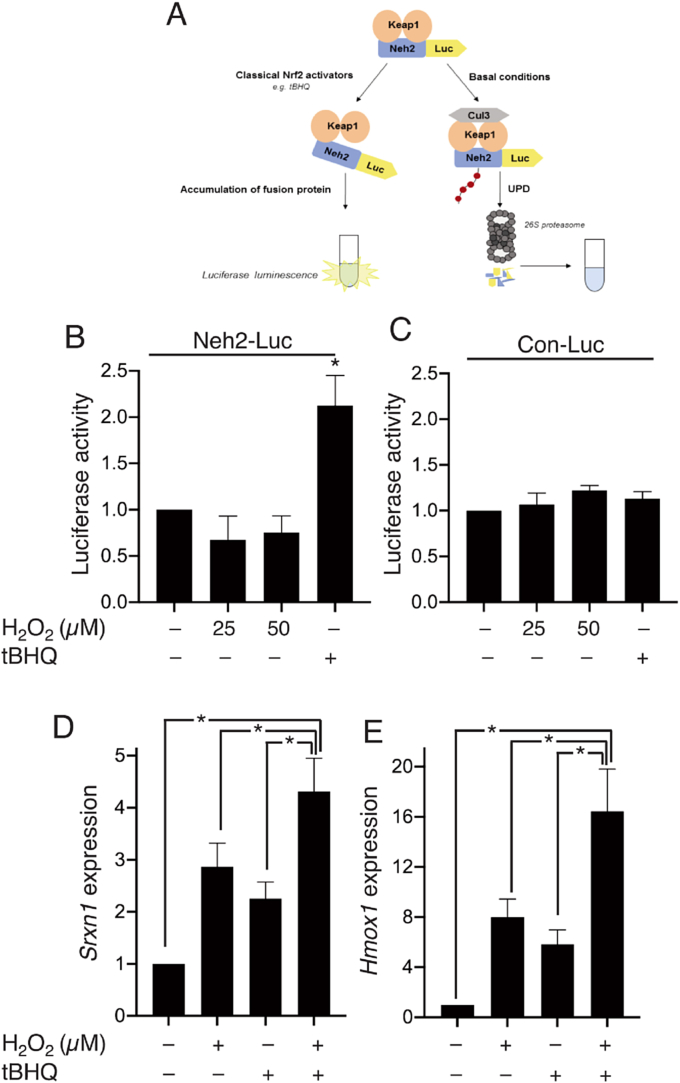
**Oxidative stress and tBHQ act on Nrf2 additively via different mechanisms. A-C)** tBHQ but not H_2_O_2_ inhibits Neh2-mediated protein degradation. A) Schematic representation of the Neh2-luc reporter system. Astrocytes within mixed astrocyte/neuronal cultures were transfected with Neh2-luc (**B**) or control luciferase reporter plasmid (**C**) plus a pTK-renilla plasmid. The approach to target astrocyte transfection within mixed neuronal/astrocyte cultures was described previously [38,42,43] and verified for this study (Figs. S3a and b). Treatment with tBHQ or H_2_O_2_ for 8h was carried out 6 days post transfection (DIV08) before assessing luciferase reporter activity. Firefly luciferase expression was normalized to Renilla luciferase control. *P < 0.05, two-way ANOVA with Tukey's *post-hoc* test, (n = 3–4), Con-Luc, luciferase control vector. **D,E)** Mixed astrocyte/neuronal cultures were treated with H_2_O_2_ and/or tBHQ as indicated for 6h, gene expression was assessed by qRT-PCR and normalized to Gapdh. *P < 0.05, one-way ANOVA with Sidak's *post-hoc* test (n = 5–6).

Since Keap1 interacts with Nrf2's Neh2 domain, the Neh2 ‘degron’ confers instability on fusion proteins, and Neh2-luciferase was developed as a reporter of treatments that influence Neh2-dependent degradation [24] (Fig. 3a). Astrocytes were transfected with a plasmid encoding Neh2-luciferase, or a luciferase control, and treated with either tBHQ or H_2_O_2_. TBHQ significantly increased the luciferase signal in Neh2-luciferase expressing astrocytes, however, H_2_O_2_ treatment had no effect (Fig. 3b). Thus, tBHQ but not H_2_O_2,_ inhibits Neh2-dependent protein degradation, further evidence that H_2_O_2_ is inducing Nrf2-dependent gene expression via a Keap1/Neh2-independent mechanism, distinct from the canonical pathway activated by tBHQ. (As a control, we observed no effect on the non-Neh2 fused Luciferase control, Fig. 3c).A direct prediction of this is that the effect of tBHQ and H_2_O_2_ should be additive, rather than occlude each other, and indeed this is the case: combined treatment produced a larger induction of Nrf2 target genes than treatment alone (Fig. 3d and e), consistent with their distinct mechanisms of action.

**Fig. 4:**
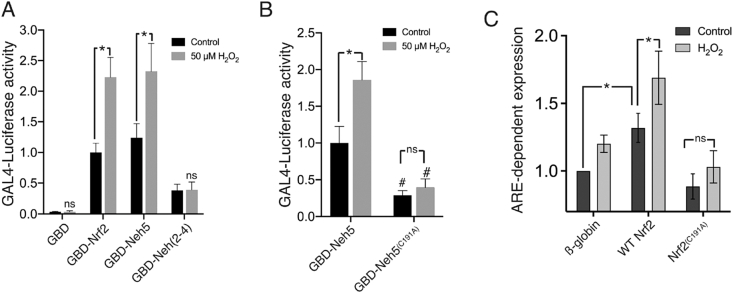
Mild oxidative stress directly regulates Nrf2 transcriptional activity through the Neh5 domain. **A)** Astrocytes within mixed astrocyte/neuronal cultures were co-transfected with Gal4-luc plasmid and either of the Nrf2-GBD fusion plasmids, plus pTK-renilla. Treatment with H_2_O_2_ (50 μM) for 8h was carried out 6 days post transfection (DIV08) before assessing luciferase reporter activity. Luciferase activity levels were normalized to Renilla and presented relative to GBD-Nrf2 control. *P < 0.05, significantly different from GBD-Nrf2 control, two-way ANOVA with Tukey's *post-hoc* test, (n = 3–10). **B)** Experiment performed as in (A) employing GBD-Neh5 (WT) and C191A mutant. *P < 0.05, significantly different from GBD-Neh5 control, two-way ANOVA with Tukey's *post-hoc* test, main effect of mutation was detected (GBD-Neh5-control Vs GDB-Neh5(C191A)) (two-way ANOVA, *P < 0.05), (n = 3–9). **C)** Astrocytes within mixed astrocyte/neuronal cultures were co-transfected with an ARE-dependent luciferase reporter, plus either WT or mutant Nrf2, or β-globin control, then stimulated ± H_2_O_2_ for 8h Luciferase activity levels were normalized to Renilla and presented relative to the unstimulated β-globin control (which relies on only endogenous Nrf2 to drive the reporter). *P < 0.05 two-way ANOVA, with Sidak's *post-hoc* test (n = 4).

An alternative, Keap1-independent mechanism of action by which H_2_O_2_ activates Nrf2-dependent gene expression could involve direct regulation of Nrf2's transactivation potential, which is mediated by two domains at the N-terminus, Neh5 and Neh4 [25]. We employed fusion proteins of the Gal4 DNA binding domain (GBD) to full length Nrf2 (GBD-Nrf2), amino-acids 1–156 (GBD-Neh2-4), and amino acids 153–227 (GBD-Neh5). All fusion proteins enhanced expression of a Gal4-luciferase reporter, relative to expression of the Gal4 DBD alone (Fig. 4a), although Neh5 was particularly potent, particularly as it appeared to be expressed at lower levels than GBD-Neh2-4 (Figs. S4a and b). Of note, H_2_O_2_ application induced reporter expression mediated by GBD-Nrf2 and by GBD-Neh5, but not by GBD-Neh2-4 (Fig. 4a). Collectively, these observations are consistent with induction of Nrf2's transactivation properties by acting on its Neh5 domain.

Cysteine residues have a well-defined role in sensing and conveying changes in cellular redox status [26,27]. The human Nrf2 Neh5 domain is known to harbor a redox-sensitive cysteine residue [28] so we mutated the equivalent cysteine residue in the murine Nrf2 (Cys-191) to create GBD-Neh5^C191A^. This point mutation did not influence expression levels of the construct (Figs. S4a and b) but not only reduced the basal transactivation potential of the Neh5 domain, it also abolished its response to H_2_O_2_ (Fig. 4b), pointing to this residue as a target in the induction of Nrf2-dependent gene expression in astrocytes by mild oxidative stress. A similar loss of basal and H_2_O_2_-induced activity was observed in full length Nrf2^C191A^ compared to WT Nrf2, assaying their ability to activate an ARE reporter based on the *Srxn1* promoter [29] (Fig. 4c). Both Nrf2^C191A^ and WT Nrf2 were expressed at the same level, and showed similar subcellular localisation (Figs. S4c and d). Thus cysteine-191 plays a role in the context of the basal and H_2_O_2_-induced activity of Nrf2 without any alteration in protein stability or location.

## 3 Discussion

In this study we presented a previously unrecognized mechanism for astrocytic Nrf2 activation by mild oxidative stress that is independent of the classical Keap1 antagonism pathway, involving direct regulation of Nrf2 via the Neh5 transactivation domain. While it was assumed that Keap1 inhibition was the dominant mechanism for Nrf2 induction by oxidative stress in astrocytes, this had never been investigated until now. Overall, our data demonstrate that non-lethal oxidative stress activates astrocytic Nrf2 in no small part via a Keap1-independent manner, meaning that the effects of oxidative stress and classical electrophilic Nrf2 inducers can be additive. This is relevant from a translational point of view because it suggests that even in cells experiencing oxidative stress, that classical electrophilic Nrf2 inducers can have an Nrf2-boosting effect, rather than their effects being occluded by the pre-existing ROS generation. Interestingly, recent work has shown that low-level H_2_O_2_ production in astrocytes leads to reduced extracellular ROS by tonically activating Nrf2 [30] suggesting that the atypical mechanism of Nrf2 activation by H_2_O_2_ in astrocytes (this study) may serve a protective role in the brain.

It is important to note, however, that this non-canonical activation of Nrf2 target genes remains very different from the situation in neurons, where many Nrf2 target genes are regulated by neuronal activity in a manner completely independent of Nrf2 [[31], [32], [33]], forming part of an activity-dependent neuroprotective transcriptional program [34,35]. However, some aspects of the Keap1-independent mechanism reported here require further investigation. For instance, it will be worthwhile to study the influence of Nrf2-C191A on the endogenous expression of Nrf2-target genes and on Nrf2 cellular distribution given that Neh5 also possesses a redox-sensitive nuclear export sequence [28]. Moreover, the Keap1-independency of human astrocytes (including iPSC-derived astrocytes), increasingly used to study Nrf2 activation and influence on neurotoxicity [15,36,37], should be confirmed. To conclude, oxidative stress is a complex event and its control of Nrf2 activity is expected to be intricate involving regulation at the cellular and molecular level. Although oxidative stress activates Nrf2 independent of cell type, the underlying mechanism could be cell type-specific and may be influenced by the intensity and the duration of the insult.

## 4 Materials and methods

### 4.1 Primary cortical cultures

Cells were prepared from E17.5 wild-type, Nrf2^−/−^ and Keap1^−/−^ mice embryos as previously described [12,38,39] from Nrf2^−/−^ and Keap1^−/−^ mice, originally developed by Prof. M. Yamamoto laboratory (University of Tohoku) [3,40] were obtained from Prof. John. D. Hayes (University of Dundee). However, Nrf2^−/−^ mice have been backcrossed over six generations onto C57BL/6 genetic background [41]. Offspring of Nrf2^−/−^ mice was generated through breeding of Nrf2^−/−^ females and males. Matching C57BL/6 WT animals were used to generate parallel wild-type cultures. Keap1 heterozygote males and females were mated to produce Keap1^+/+^ and Keap1^−/−^ littermates which were used for comparison.

### 4.2 Statistical analysis

All results were obtained from at least three biological replicates (defined as independently performed experiments on material from cortical cultures derived from different animals) and within each experiment 2 or 3 technical replicates were included per condition. Data is presented as mean ± standard error of the mean. Statistical testing was carried out using Student's t-test. For studies employing multiple testing one or two-way analysis of variance (ANOVA) was applied.

### 4.3 Other methods

See Supplemental Methods for details of induction of stress and assessment of cell viability, of plasmids and primers used, plus details of luciferase reporter assays, qPCR and RNA-seq.

## Funding

This work was funded by the 10.13039/501100017510UK Dementia Research Institute which receives its funding from UK DRI Ltd, itself funded by the 10.13039/100007472UK 10.13039/501100000265Medical Research Council, Alzheimer's Society, and Alzheimer's Research UK**.**

## Author contributions

Study conceptualization: GEH; Methodology: GEH, IG; Investigation/Experimentation: BRA, KFSB, SC, PJM, PSB, SM; Data Analysis, GEH, BRA, OD; Resources: IG, JDH, MLJA; Writing – Original Draft Preparation, BRA; Writing – Review & Editing, GEH, JDH; Funding Acquisition; GEH, JDH.

## Declaration of competing interest

The authors declare that they have no known competing financial interests or personal relationships that could have appeared to influence the work reported in this paper.

## References

[1] Ishii T., Itoh K., Yamamoto M. (2002). Roles of Nrf2 in activation of antioxidant enzyme genes via antioxidant responsive elements. Methods Enzymol..

[2] Rushmore T.H., Morton M.R., Pickett C.B. (1991). The antioxidant responsive element. Activation by oxidative stress and identification of the DNA consensus sequence required for functional activity. J. Biol. Chem..

[3] Itoh K. (1997). An Nrf2/small Maf heterodimer mediates the induction of phase II detoxifying enzyme genes through antioxidant response elements. Biochem. Biophys. Res. Commun..

[4] Itoh K. (1999). Keap1 represses nuclear activation of antioxidant responsive elements by Nrf2 through binding to the amino-terminal Neh2 domain. Genes Dev..

[5] Yamamoto M., Kensler T.W., Motohashi H. (2018). The KEAP1-NRF2 system: A Thiol-based Sensor-Effector Apparatus for Maintaining redox Homeostasis. Physiol. Rev..

[6] Johnson D.A., Johnson J.A. (2015). Nrf2--a therapeutic target for the treatment of neurodegenerative diseases. Free Radic. Biol. Med..

[7] Brandes M.S., Gray N.E. (2020).

[8] Sigfridsson E. (2018). Astrocyte-specific overexpression of Nrf2 protects against optic tract damage and behavioural alterations in a mouse model of cerebral hypoperfusion. Sci. Rep..

[9] Johnson D.A., Andrews G.K., Xu W., Johnson J.A. (2002). Activation of the antioxidant response element in primary cortical neuronal cultures derived from transgenic reporter mice. J. Neurochem..

[10] Kraft A.D., Johnson D.A., Johnson J.A. (2004). Nuclear factor E2-related factor 2-dependent antioxidant response element activation by tert-butylhydroquinone and sulforaphane occurring preferentially in astrocytes conditions neurons against oxidative insult. J. Neurosci..

[11] Murphy T.H. (2001). Preferential expression of antioxidant response element mediated gene expression in astrocytes. J. Neurochem..

[12] Bell K.F.S. (2015). Neuronal development is promoted by weakened intrinsic antioxidant defences due to epigenetic repression of Nrf2. Nat. Commun..

[13] Jimenez-Blasco D., Santofimia-Castano P., Gonzalez A., Almeida A., Bolanos J.P. (2015). Astrocyte NMDA receptors' activity sustains neuronal survival through a Cdk5-Nrf2 pathway. Cell Death Differ..

[14] Shih A.Y. (2003). Coordinate regulation of glutathione biosynthesis and release by Nrf2-expressing glia potently protects neurons from oxidative stress. J. Neurosci..

[15] Gupta K. (2012). Human embryonic stem cell derived astrocytes mediate non-cell-autonomous neuroprotection through endogenous and drug-induced mechanisms. Cell Death Differ..

[16] Bell K.F. (2011). Mild oxidative stress activates Nrf2 in astrocytes, which contributes to neuroprotective ischemic preconditioning. Proc. Natl. Acad. Sci. U. S. A..

[17] Yang T. (2020). Ischemic preconditioning provides long-lasting neuroprotection against ischemic stroke: The role of Nrf2. Exp. Neurol..

[18] Malhotra D. (2010). Global mapping of binding sites for Nrf2 identifies novel targets in cell survival response through ChIP-Seq profiling and network analysis. Nucleic Acids Res..

[19] Hasel P. (2017). Neurons and neuronal activity control gene expression in astrocytes to regulate their development and metabolism. Nat. Commun..

[20] Qiu J. (2018). Mixed-species RNA-seq for elucidating non-cell-autonomous control of gene transcription. Nat. Protoc..

[21] Zhang D.D., Hannink M. (2003). Distinct cysteine residues in Keap1 are required for Keap1-dependent ubiquitination of Nrf2 and for stabilization of Nrf2 by chemopreventive agents and oxidative stress. Mol. Cell Biol..

[22] Abiko Y., Miura T., Phuc B.H., Shinkai Y., Kumagai Y. (2011). Participation of covalent modification of Keap1 in the activation of Nrf2 by tert-butylbenzoquinone, an electrophilic metabolite of butylated hydroxyanisole. Toxicol. Appl. Pharmacol..

[23] Zhang D.D. (2006). Mechanistic studies of the Nrf2-Keap1 signaling pathway. Drug Metab. Rev..

[24] Smirnova N.A. (2011). Development of Neh2-luciferase reporter and its application for high throughput screening and real-time monitoring of Nrf2 activators. Chem. Biol..

[25] Katoh Y. (2001). Two domains of Nrf2 cooperatively bind CBP, a CREB binding protein, and synergistically activate transcription. Gene Cell..

[26] Barford D. (2004). The role of cysteine residues as redox-sensitive regulatory switches. Curr. Opin. Struct. Biol..

[27] Reth M. (2002). Hydrogen peroxide as second messenger in lymphocyte activation. Nat. Immunol..

[28] Li W., Yu S.W., Kong A.N. (2006). Nrf2 possesses a redox-sensitive nuclear exporting signal in the Neh5 transactivation domain. J. Biol. Chem..

[29] Papadia S. (2008). Synaptic NMDA receptor activity boosts intrinsic antioxidant defenses. Nat. Neurosci..

[30] Vicente-Gutierrez C. (2019). Astrocytic mitochondrial ROS modulate brain metabolism and mouse behaviour. Nat Metab.

[31] Lewerenz J. (2014). Phosphoinositide 3-kinases upregulate system xc(-) via eukaryotic initiation factor 2alpha and activating transcription factor 4 - A pathway active in glioblastomas and epilepsy. Antioxidants Redox Signal..

[32] Deighton R.F. (2014). Nrf2 target genes can be controlled by neuronal activity in the absence of Nrf2 and astrocytes. Proc. Natl. Acad. Sci. U. S. A..

[33] Soriano F.X. (2009). Transcriptional regulation of the AP-1 and Nrf2 target gene sulfiredoxin. Mol. Cell..

[34] Baxter P.S., Martel M.A., McMahon A., Kind P.C., Hardingham G.E. (2011). Pituitary adenylate cyclase-activating peptide induces long-lasting neuroprotection through the induction of activity-dependent signaling via the cyclic AMP response element-binding protein-regulated transcription co-activator 1. J. Neurochem..

[35] Bell K.F., Hardingham G.E. (2011). The influence of synaptic activity on neuronal health. Curr. Opin. Neurobiol..

[36] Gupta K., Hardingham G.E., Chandran S. (2013). NMDA receptor-dependent glutamate excitotoxicity in human embryonic stem cell-derived neurons. Neurosci. Lett..

[37] Gupta K., Chandran S., Hardingham G.E. (2012). Human stem cell-derived astrocytes and their application to studying Nrf2-mediated neuroprotective pathways and therapeutics in neurodegeneration. Br. J. Clin. Pharmacol..

[38] Puddifoot C. (2012). PGC-1alpha negatively regulates extrasynaptic NMDAR activity and excitotoxicity. J. Neurosci..

[39] Al-Mubarak B., Soriano F.X., Hardingham G.E. (2009). Synaptic NMDAR activity suppresses FOXO1 expression via a cis-acting FOXO binding site: FOXO1 is a FOXO target gene. Channels (Austin, Tex.

[40] Wakabayashi N. (2003). Keap1-null mutation leads to postnatal lethality due to constitutive Nrf2 activation. Nat. Genet..

[41] Higgins L.G. (2009). Transcription factor Nrf2 mediates an adaptive response to sulforaphane that protects fibroblasts in vitro against the cytotoxic effects of electrophiles, peroxides and redox-cycling agents. Toxicol. Appl. Pharmacol..

[42] Alabdullah A.A. (2019). Estimating transfection efficiency in differentiated and undifferentiated neural cells. BMC Res. Notes.

[43] Marwick K.F.M., Hardingham G.E. (2017). Transfection in primary cultured neuronal cells. Methods Mol. Biol..

