# Supplementary material for: Non-canonical Keap1-independent activation of Nrf2 in astrocytes by mild oxidative stress
Source: Redox Biol. 2021 Oct 2;47:102158. doi: 10.1016/j.redox.2021.102158 (PMC8512624; doi:10.1016/j.redox.2021.102158)
Supplement: Multimedia component 1 [file mmc1.docx]

**Supplemental Materials and Methods**

**Primary cortical cultures**

Astrocyte cultures, mixed neuron/astrocyte cultures and astrocyte-free neuronal cultures were prepared as previously described ^1-3^. For mixed-species co-cultures, rat neurons were plated on top of a confluent layer of DIV14 mouse astrocytes and both astrocyte mono-cultures and astrocyte-neuron co-cultures were subsequently kept in Neurobasal-A medium containing B27 (both Life Technologies), but devoid of serum. Note that the rat neuronal cells plated do contain a small number of non-neuronal cells (principally astrocytes), but since the focus of the study is the mouse astrocytic transcriptome, this was deemed acceptable. Genotype/age use was E17.5 wild-type, Nrf2^-/-^ and Keap1^-/-^ mice embryos, originally developed by Prof. M. Yamamoto laboratory (University of Tohoku) ^4,5^. Nrf2^-/-^ mice have been backcrossed over six generations onto C57BL/6 genetic background ^6^. Offspring of Nrf2^-/-^ mice was generated through breeding of Nrf2^-/-^ females and males. Matching C57BL/6 WT animals were used to generate parallel wild-type cultures. Keap1 heterozygote males and females were mated to produce Keap1^+/+^ and Keap1^-/-^ littermates which were used for comparison. The offspring genotypes were verified through multiplex PCR analysis of cerebellum genomic DNA using DNA easy blood and tissue kit (Qiagen UK, Crawley, UK). The primers used are listed in (Table S2) and the PCR conditions were as follows: 5 minutes at 95°C followed by 34 cycles of 94°C for 30 seconds, 55°C for 1 minute and 72°C for 1 minute, followed by an extension step at 72°C for 10 minutes. The size of the bands produced by this reaction are distinctive based on the genotype of the sample DNA whereby Keap1 WT and KO samples generate bands of 235 and 420 base pairs (bp) respectively.

**Table S2. Genotyping primers**

| PCR Primer | Sequence |
| --- | --- |
| D123 | 5’-CGGGATCCCCATGGAAAGGCTTATTGAGTTC-3’ |
| 2nd Ex 3’ | 5’-GAAGTGCATGTAGATATACTCCC-3’ |
| Tv Neo | 5’-TCAGAGCAGCCGATTGTGTGTTGTGCCCAGTCA-3’ |

**Application of oxidative stressors and Nrf2 activators**

Before treatment, cells were incubated in serum-free trophically deprived transfection medium (TMo) for 2-4h containing 10% minimum essential media (Life Technologies Ltd) and 90% Salt-Glucose-Glycine (SGG) medium, which is comprised of 114mM NaCl, 0.219% NaHCO3, 5.292mM KCl, 1mM MgCl2, 2mM CaCl2, 10mM HEPES, 1mM Glycine, 30mM Glucose, 0.5mM sodium pyruvate, 0.1% Phenol Red. Working stock of tBHQ (Sigma-Aldrich) was prepared at a concentration of 5mM in DMSO in aliquots that were stored at -20°C until needed. At the time of experiment tBHQ was added at a final concentration of 10μM. Working stock of hydrogen peroxide solution (3%w/w, equivalent to 880mM, Sigma-Aldrich), was prepared at 8.8mM in dH_2_O on the same day of the experiment.

**Assessment of cell viability**

For cell death quantification, mixed astrocyte/neuronal cultures were fixed with 4% paraformaldehyde (PFA) and subjected to 4’,6’ diamidino-2-phenylindole (DAPI) (Vector Laboratories, California, USA) nuclear staining and cell death was quantified by counting the number of pyknotic nuclei as a percentage of the total. Approximately 1500 cells were scored across several random fields within 3-4 independent experiments.

**Transient transfections and plasmids**

All transfections were performed using Lipofectamine 2000 (Invitrogen) on cells cultured in 24-well plates. We applied a protocol that preferentially targets the astrocytic population in our cultures ^7,8^. Briefly, on DIV2 cells were transferred to TMo 2-4h prior to transfection. For each well, a DNA (µg): Lipofectamine (µl) ratio of (1:3.88) was applied and the cells were incubated with transfection complexes for 3-4h before they were returned to growth medium. Cells were maintained in culture for 6 days (DIV8) before experimentation.

For the GBD-Neh5 and pEF-Nrf2 mutation studies, the reactive cysteine residue 191 (Cys-191) was substituted with alanine. To obtain this, QuikChange II XL site-directed mutagenesis kit (Stratagene) was used along with the mutagenic primer and its reverse complementary sequence containing the mismatches 5′-CATTCCCGAATTA CAG**GCC**CT TAATACCGAAAACAAG-3′ (bold indicates nucleotides changed, underlined sequence indicates creation of EcoO109I diagnostic site). As for pGFP-Nrf2 mutant version, the corresponding cysteine residue in the human transcript (Cys-199) was substituted with alanine as well using the same kit and the following mutagenic primer and its reverse complementary sequence containing the mismatches 5’-CGGAGTTACAG**GC**TCT**G**AATATTGAAAATGACAAGC-3’ (bold indicates nucleotides changed, underlined sequence indicates creation of AlwNI diagnostic site). All mutants were verified by performing diagnostic cuts with the indicated restriction enzymes and were further confirmed by sequencing (SBS sequencing service, University of Edinburgh). A list of all the plasmids used in this study are shown below (Table S3)

**Table S3. List of plasmids used in this study**

| **Plasmid name** | **Description** | **Reference** |
| --- | --- | --- |
| pGL2-SV40-Luc | SV40-driven empty Luciferase reporter control | Promega |
| pTK-RL | Thymidine kinase promoter driven Renilla luciferase reporter | Promega |
| Gal4-Luc | Luciferase reporter bearing 4 copies of Gal4-binding site | Promega |
| GBD-Nrf2 | Gal4 DNA binding domain fused to the full length mouse Nrf2 | - ^9^ |
| GBD-Neh5 | Gal4 DNA binding domain fused to Neh5 domain (153-227 amino acids) |  |
| GBD-Neh(2-4) | Gal4 DNA binding domain fused to both Neh2 and Neh4 domains (1-156 amino acids) |  |
| GBD | Gal4 DNA binding domain only |  |
| ARE-Luc | ARE-containing portion of the Srxn1 promoter regulating luciferase expression | ^10^ |
| Neh2-Luc | Neh2 domain fused to luciferase gene (Neh2-Luc) | ^11^ |
| pEF-Nrf2 | mouse full length Nrf2 was cloned into pEF1-α neo expression vector | ^12^ |
| pGFP-Nrf2 | human full length Nrf2 was cloned into pGFP/pOB125 expression vector | ^13^ |

**Luciferase reporter gene assay**

Luciferase assays were carried out on cells transfected with the desired constructs at the following DNA ratio 2:1:4 for luciferase-reporter: Tk-Renilla (normalization control): effector plasmid. For the Gal4-based reporter assay, transfections were carried out using the reporter plasmid Gal4-Luc and pTK-renilla and either of the effector plasmids; GBD-Nrf2, GBD-Neh(2-4), GBD-Neh5 or GBD only. As for the Neh2-Luciferase reporter system, cells were transfected with either Neh2-Luc or Con-Luc along with pTK-renilla in the following proportion 4:1 of reporter gene to pTK-renilla. For the ARE-Luc experiments, the DNA ratio was 4:1:1 for luciferase-reporter: Tk-Renilla (normalization control): effector plasmid (WT or mutant pEF-Nrf2). To look at WT vs mutant Nrf2 localization, pGFP-Nrf2 or the S191A mutant was transfected and cells fixed 48h later.

**RNA extraction and qPCR**

Total RNA was isolated using High Pure RNA Isolation Kit (Roche, Welwyn Garden City,UK) including a DNase-treatment step to degrade genomic DNA. cDNA was synthesized from 1-5μg RNA using Transcriptor First Strand cDNA Synthesis Kit (Roche). Each qPCR reaction contained 6 ng of cDNA mixed with FS Universal SYBR Green MasterRox (Roche) and was carried out in an Mx3000P qPCR system (Agilent Technologies, Cheshire, UK). In each experiment, technical duplicates were used for every sample including NoRT and No-template controls. The cycling parameters were as follows: 10 minutes of initial denaturation at 95°C; 40 cycles of 30 seconds at 95°C, 40 seconds of annealing at 60°C with detection of fluorescence and 30 seconds of extension at 72°C; followed by one cycle of 1 minute at 95°C, the temperature was ramped from 55°C to 95°C over 30 seconds at 1°C per step with continuous fluorescence detection (for dissociation curve analysis to confirm the amplification of a single product). Gene of interest expression was normalized to Gapdh levels and subsequently compared to levels in control samples using the 2(-ΔΔCt) efficiency corrected method ^14^. Q-PCR primer sequences used in this study (Table S4)

**Table S4. List of all the qPCR used in these studies.**

| - **Gene** | - **Forward primer (5′-3′)** | - **Reverse primer (5′-3′)** |
| --- | --- | --- |
| - *Gapdh* | - GGGTGTGAACCACGAGAAT | - CCTTCCACAATGCCAAAGTT |
| - *Slc7a11/xCT* | - ATACTCCAGAACACGGGCAG | - AGTTCCACCCAGACTCGAAC |
| - *Srxn1* | - GACGTCCTCTGGATCAAAG | - GCAGGAATGGTCTCTCTCTG |
| - *Hmox1* | AGCACAGGGTGACAGAAGAG | - GGAGCGGTGTCTGGGATG |

**RNA-seq and Species-specific sorting of mixed species reads**

To generate RNA-seq data, barcoded RNA-seq libraries were prepared by Edinburgh Genomics using the Illumina TruSeq stranded mRNA-seq kit, according to the manufacturer’s protocol (Illumina). The libraries were pooled and sequenced to 50 base paired-end on an Illumina NovaSeqTM 6000. For single-species RNA-seq experiments sequencing was performed to a depth of approximately 50 million paired-end reads per sample, whereas for mixed species RNA-seq a greater depth of approximately 150 million paired-end reads per sample was done. Species-specific separation of RNA-seq reads was performed using version 1.2 of Sargasso ^15^ (during which reads were mapped to the mouse, rat and human genomes using version 2.7.0 of STAR ^16^). The protocol is described in detail elsewhere ^15^. Subsequently, per-gene read counts were summarised using featureCounts version 1.6.3 ^17^. For read mapping and feature counting, genome sequences and gene annotations were downloaded from Ensembl version 101. Differential expression (DGE) analysis on data sets was performed using DESeq2 ^18^ (R package version 1.24.0) using a significance threshold set at a Benjamini-Hochberg-adjusted p-value of 0.05. Before arriving at a final DGE dataset for the microglia, we carried out an additional control (as recommended in our published protocol ^15^) by performing RNA-seq on a two-species co-culture of mouse neurons and human astrocytes, and determining whether the Sargasso workflow resulted in any human or mouse reads being incorrectly called as rat. We took a conservative approach and discarded any genes for which we estimated >10% of rat reads within the mixed species co-culture could be due to incorrectly called human or mouse reads.

**References**

1 Puddifoot, C. *et al.* PGC-1alpha negatively regulates extrasynaptic NMDAR activity and excitotoxicity. *J Neurosci* **32**, 6995-7000, doi:32/20/6995 [pii]

10.1523/JNEUROSCI.6407-11.2012 (2012).

2 Bell, K. F. S. *et al.* Neuronal development is promoted by weakened intrinsic antioxidant defences due to epigenetic repression of Nrf2. *Nature Communications* **6**, 7066, doi:10.1038/ncomms8066 (2015).

3 Al-Mubarak, B., Soriano, F. X. & Hardingham, G. E. Synaptic NMDAR activity suppresses FOXO1 expression via a cis-acting FOXO binding site: FOXO1 is a FOXO target gene. *Channels (Austin, Tex* **3**, 233-238, doi:9381 [pii] (2009).

4 Itoh, K. *et al.* An Nrf2/small Maf heterodimer mediates the induction of phase II detoxifying enzyme genes through antioxidant response elements. *Biochem Biophys Res Commun* **236**, 313-322, doi:S0006291X97969436 [pii] (1997).

5 Wakabayashi, N. *et al.* Keap1-null mutation leads to postnatal lethality due to constitutive Nrf2 activation. *Nat Genet* **35**, 238-245, doi:10.1038/ng1248 (2003).

6 Higgins, L. G. *et al.* Transcription factor Nrf2 mediates an adaptive response to sulforaphane that protects fibroblasts in vitro against the cytotoxic effects of electrophiles, peroxides and redox-cycling agents. *Toxicol Appl Pharmacol* **237**, 267-280, doi:S0041-008X(09)00115-X [pii]

10.1016/j.taap.2009.03.005 (2009).

7 Alabdullah, A. A. *et al.* Estimating transfection efficiency in differentiated and undifferentiated neural cells. *BMC Res Notes* **12**, 225, doi:10.1186/s13104-019-4249-5 (2019).

8 Marwick, K. F. M. & Hardingham, G. E. Transfection in Primary Cultured Neuronal Cells. *Methods Mol Biol* **1677**, 137-144, doi:10.1007/978-1-4939-7321-7_6 (2017).

9 Katoh, Y. *et al.* Two domains of Nrf2 cooperatively bind CBP, a CREB binding protein, and synergistically activate transcription. *Genes Cells* **6**, 857-868, doi:469 [pii] (2001).

10 Papadia, S. *et al.* Synaptic NMDA receptor activity boosts intrinsic antioxidant defenses. *Nat Neurosci* **11**, 476-487 (2008).

11 Smirnova, N. A. *et al.* Development of Neh2-luciferase reporter and its application for high throughput screening and real-time monitoring of Nrf2 activators. *Chem Biol* **18**, 752-765, doi:S1074-5521(11)00164-5 [pii]

10.1016/j.chembiol.2011.03.013 (2011).

12 Kotkow, K. J. & Orkin, S. H. Dependence of globin gene expression in mouse erythroleukemia cells on the NF-E2 heterodimer. *Mol Cell Biol* **15**, 4640-4647 (1995).

13 Numazawa, S., Ishikawa, M., Yoshida, A., Tanaka, S. & Yoshida, T. Atypical protein kinase C mediates activation of NF-E2-related factor 2 in response to oxidative stress. *American journal of physiology. Cell physiology* **285**, C334-342 (2003).

14 Livak, K. J. & Schmittgen, T. D. Analysis of relative gene expression data using real-time quantitative PCR and the 2(-Delta Delta C(T)) Method. *Methods* **25**, 402-408, doi:10.1006/meth.2001.1262 (2001).

15 Qiu, J. *et al.* Mixed-species RNA-seq for elucidating non-cell-autonomous control of gene transcription. *Nat Protoc* **13**, 2176-2199, doi:10.1038/s41596-018-0029-2 (2018).

16 Dobin, A. *et al.* STAR: ultrafast universal RNA-seq aligner. *Bioinformatics* **29**, 15-21, doi:bts635 [pii]

10.1093/bioinformatics/bts635 (2013).

17 Liao, Y., Smyth, G. K. & Shi, W. featureCounts: an efficient general purpose program for assigning sequence reads to genomic features. *Bioinformatics* **30**, 923-930, doi:btt656 [pii]

10.1093/bioinformatics/btt656 (2014).

18 Love, M. I., Huber, W. & Anders, S. Moderated estimation of fold change and dispersion for RNA-seq data with DESeq2. *Genome Biol* **15**, 550, doi:s13059-014-0550-8 [pii]

10.1186/s13059-014-0550-8 (2014).
