# Supplementary material for: Non-canonical Keap1-independent activation of Nrf2 in astrocytes by mild oxidative stress
Source: Redox Biol. 2021 Oct 2;47:102158. doi: 10.1016/j.redox.2021.102158 (PMC8512624; doi:10.1016/j.redox.2021.102158)
Supplement: Multimedia component 3 [file mmc3.pdf]

Supplemental Figures

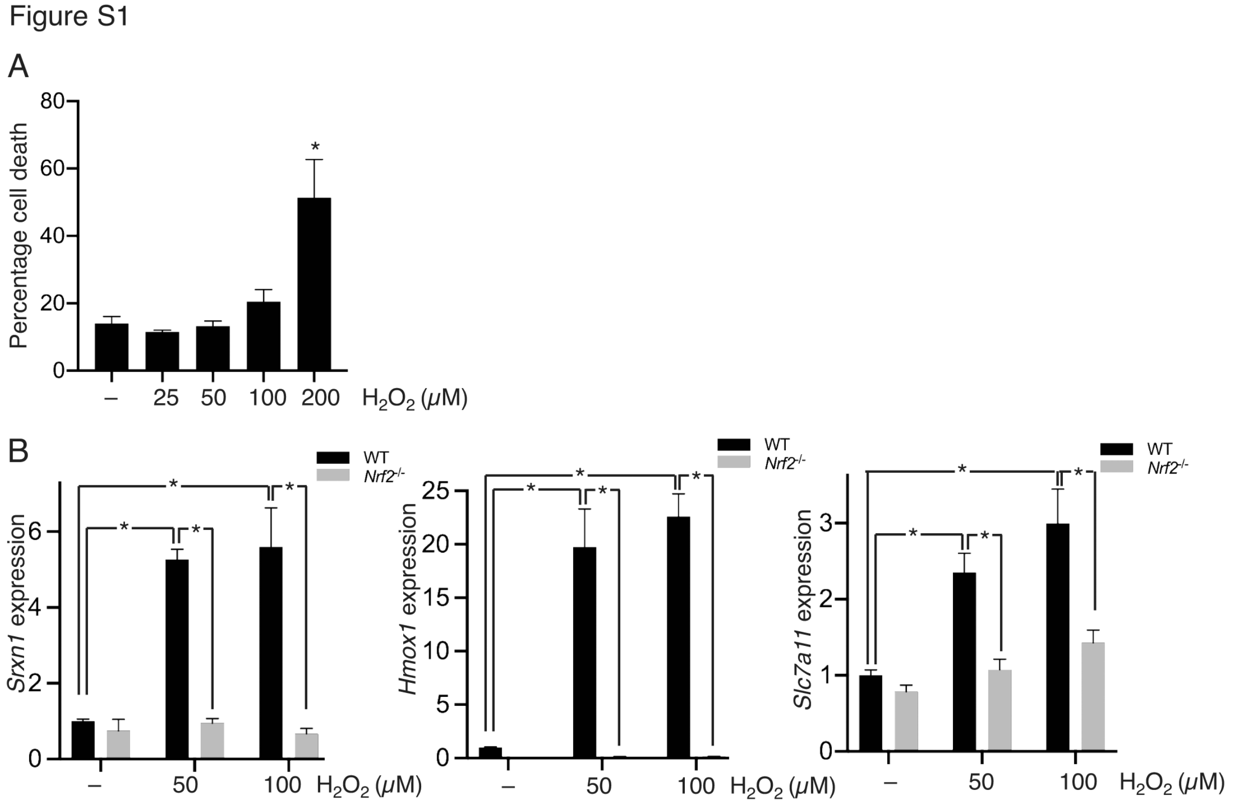

Figure S1. **A)** Cell viability in response to a range of H_2O_2 doses. To induce oxidative stress a range of H_2O_2 doses (25µM-200µM) was utilized and cell viability was evaluated in mixed astrocyte/neuronal cultures by assessing nuclear integrity. **P*<0.05, one-way ANOVA with Dunnett's *post-hoc test*, (n=3-4). **B)** H_2O_2 treatments triggers induction of *Srxn1*, *Hmox1* and *Slc7a11* expression which is eliminated by Nrf2-deficiency. Mixed astrocyte/neuronal cultures of the indicated genotypes were treated with the indicated doses of H_2O_2 for 6 h, before performing qPCR analysis of gene expression (normalized to Gapdh). Expression levels are expressed relative to WT-control. **P*<0.05, two-way ANOVA with Tukey's *post-hoc* test (n=3-9).

Figure S2

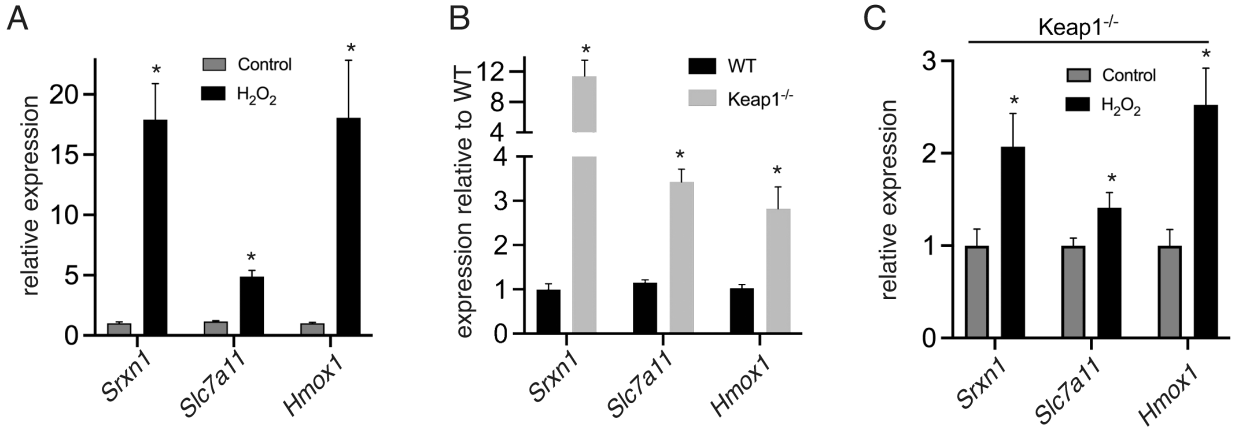

Figure S2. A) Mono-cultures of astrocytes were treated ± 100 µM H_2O_2 for 8h and the indicated genes analyzed by qPCR. **P*<0.05 (unpaired t-test, n=4). **B)** RNA was extracted from mono-cultures of astrocytes from Keap1 KO mice and WT litter mates, and the indicates genes analysed by qPCR **P*<0.05 (unpaired t-test, n=9-11). C) Keap1 KO astrocyte mono-cultures were treated as in (A)p and the indicated genes analyzed. **P*<0.05 (unpaired t-test, n=9-11).

Figure S3

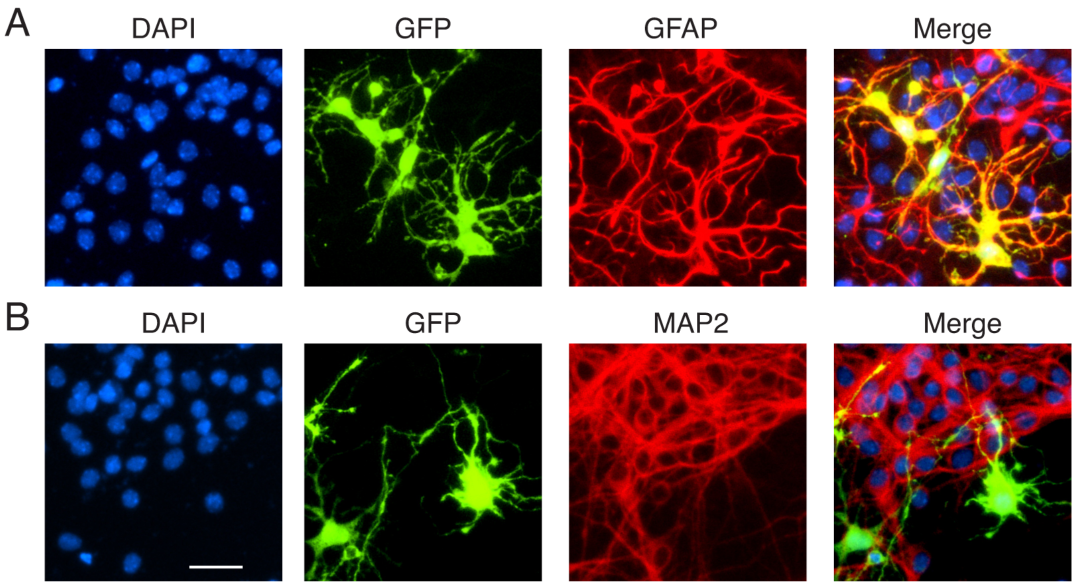

Figure S3 A,B) Example images of the approach to target astrocyte transfection within mixed neuronal/astrocyte cultures as described previously (Alabdullah et al., 2019; Marwick and Hardingham, 2017). GFAP is used as an astrocyte marker and MAP2 a neuronal marker Scale bar: 50 µm.

Figure S4

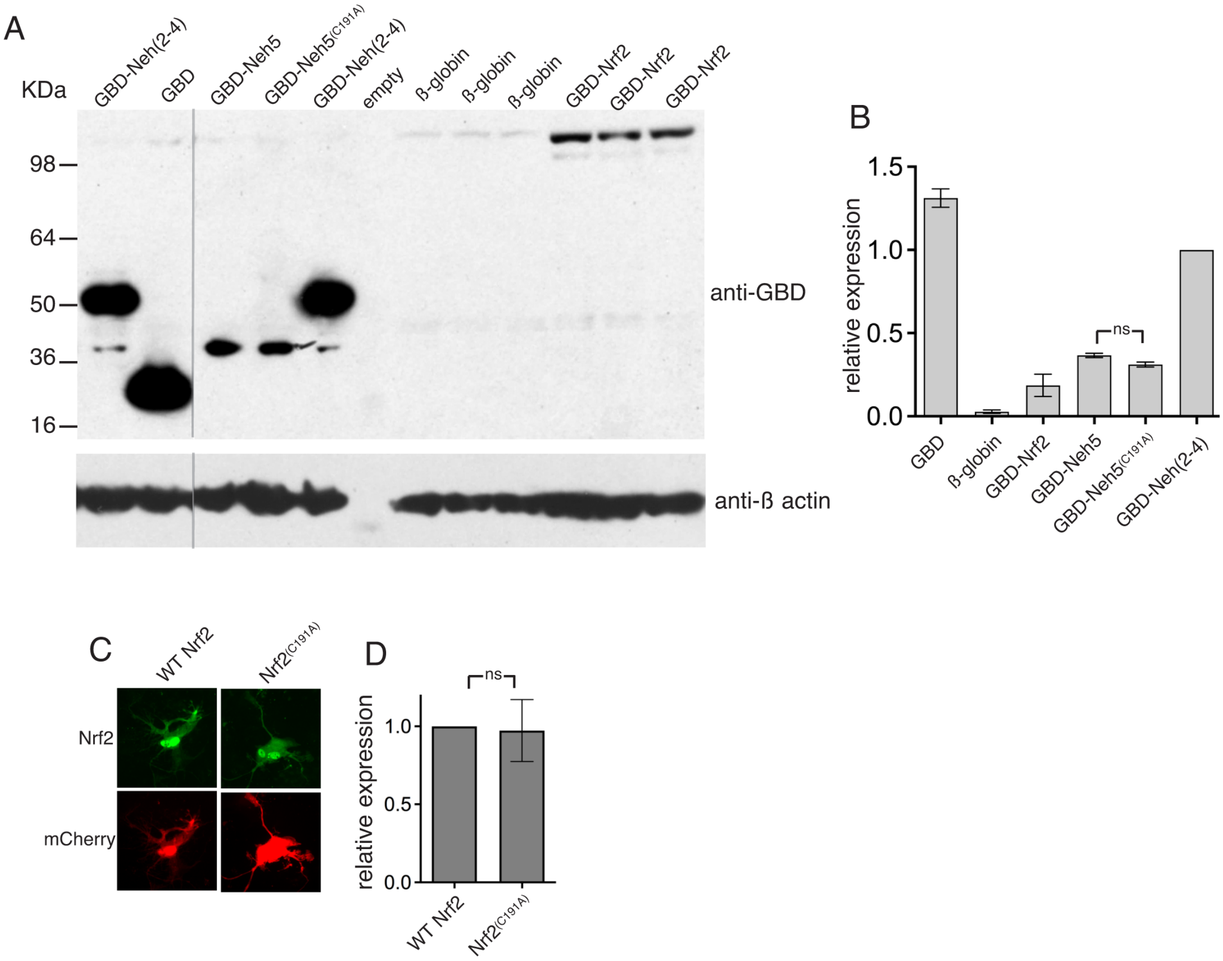

Figure S4. A,B) Constructs encoding the GAL4 DNA binding domain (GBD) fused to the indicated domains of Nrf2 were transfected into HEK-293 cells. At 24 h post transfection, protein was harvested and analyzed for GBD expression normalized to a β-actin loading control, (n=5). C,D) Astrocytes were transfected with constructs encoding WT or mutant Nrf2 tagged with GFP, plus a mCherry transfection marker. Levels of ectopically expressed Nrf2 were quantified in 78 (WT) and 73 (mutant) transfected cells using an anti-GFP antibody (n=4).
